# Knowledge and Attitude Toward Organ Donation Among the Adult Population in Jazan, Saudi Arabia

**DOI:** 10.7759/cureus.27002

**Published:** 2022-07-19

**Authors:** Mohammed Somaili, Alanoud Masmali, Ibrahim Haqawi, Manal Al-Hulaibi, Ahmed A AlHabji, Ayoub Salami, Abdulmageed A Ageel, Yasser Sultan, Alhassan Alhazemi, Fatimah Moharg, Omar Almansour, Anas E Ahmed

**Affiliations:** 1 Internal Medicine, Jazan University, Jazan, SAU; 2 Medicine and Surgery, Jazan University, Jazan, SAU; 3 Medicine, Jazan University, Jazan, SAU; 4 Faculty of Medicine, Jazan University, Jazan, SAU; 5 Internal Medicine, Qassim University, Buraidah, SAU; 6 Community Medicine, Jazan University, Jazan, SAU

**Keywords:** saudi arabia, jazan, organ donation, attitude, knowledge

## Abstract

Background

Organ transplantation is recognized as a life-saving procedure for patients with potentially terminal illnesses. However, the population's awareness of organ donation and related issues is variable throughout the world.

Objectives

The study purposes to evaluate the knowledge and attitude toward organ donation among the adult population in the Jazan region, Saudi Arabia.

Methods

We conducted a cross-sectional questionnaire-based study on the general population in Jazan using the convenient sampling method. The questionnaire was composed of 21 items distributed over demographic characteristics, knowledge, and attitude domains. The knowledge and attitude domain levels were categorized into low, moderate, and high based on the scoring of its items. The data had been analyzed using SPSS software version 23 (IBM Corp., Armonk, NY). Frequency and percentages were used to display categorical variables. Mean and standard deviation was used to present numerical variables. The independent t-test and analysis of variance (ANOVA) test were both used to test for factors associated with knowledge score and attitude score toward organ donation.

Results

A total of 1019 participants were included in the study. The majority were between the ages of 18 and 30 years and 3.4% of them were older than 50 years. Eighty percent of participants had a university level of education or higher and 48% were students. This survey showed that 493 (48.4%) had a moderate knowledge level of organ donation (total score between 50% and 75%) with younger age, being a student, and residence status associated with a higher level of knowledge while gender and the education levels were not. Five hundred one (49.2%) of the participants reported being in agreement with organ donation and 56 (5.5%) of them reported disagreement. Most of the participants exhibited a low positive attitude toward organ donation, with 592 (58.1%) of the participants (a total score less than 50%) (score of 4 and less). Young age and being a student were the factors associated with a positive attitude while gender, education levels, residence status, and monthly status were having no significant associations with the positive attitude toward organ donation.

Conclusion

This study concluded that study participants had a moderate level of knowledge and a low positive attitude toward organ donation. The advocacy in promoting organ donation should be increased through the use of appropriate mediums to change the attitudes and enhance the willingness of people.

## Introduction

Organ transplantation has been and is still perceived as a standard treatment for patients with end-stage organ failure around the world. Since 1954, when the first organ was transplanted, the field of organ transplantation has seen numerous accomplishments, which have led to saving and drastically improving countless lives [1]. The interest in organ donation and transplantation worldwide has risen dramatically over the most recent 20 years because of the expanding rates of non-transmittable diseases [2]. Since these metabolic problems have been associated with adverse outcomes, including organ failure and death, organ transplantation is perceived as a lifesaving procedure for patients with conceivably terminal illnesses [3-4].

In Saudi Arabia (KSA), the approximate number of donated organs is about 2-4 million as compared to the more than 20 million population in countries like the United States and Spain [5-7]. The steeply expanding need between patients who need transplantation and the availability of qualified donors is a significant reason for concern that mandates further attention [8-9]. As indicated by accessible assessments, a total of 146,993 organ donations were made all around the world in 2018 [10]. In Saudi Arabia, all efforts are made to increase the donation process by increasing the awareness level among different population classes [5,11-12]. The Saudi Center for organ transplantation is leading this process across the country and is targeting 15 donors per million population within three years through their initiatives [11-12]. The more significant part was for kidneys (1006), trailed by liver (270) and heart (96) transplantations [12].

## Materials and methods

Research objective

To evaluate the knowledge of and attitude toward organ donation among the adult population in the Jazan region and to detect the potential barriers to donating organs among the general public.

Methods

Study Design

This study is designed to be an observational cross-sectional study conducted to measure the level of knowledge and attitude toward organ donation from October 2021 until the end of April 2022.

Study Setting

The study was conducted in the Jazan region. Jazan is one of the 13 regions in Saudi Arabia, located in the southwest part of KSA. It covers an area of 11,671 square kilometers, and it is a highly populated region with an estimated total population of 1.6 million according to the 2017 census. There is a total of 21 governmental hospitals - secondary and tertiary - distributed over 17 districts that provide different health care services to the population. Nevertheless, only one tertiary hospital has the privilege to transfer potential donors and recipients to higher centers to launch the donation process [5].

Study Population

Participants who fulfill the inclusion and exclusion criteria have been involved in this study:

The inclusion criteria included mentally competent individuals, 18 years or older, who were willing to participate in this study. People who were already diagnosed with dementia or another cognitive impairment were excluded from participation in this study.

Data Collection

An electronic pre-tested self-administered questionnaire in the Arabic language was used in this study. The questionnaire was created after an extensive review of different questionnaires performed on the same topic [13-15]. The electronic self-administered questionnaire was tested by a group of interviewers before implementation. The questionnaire consists of a total of 21 questions distributed over three domains. The socioeconomic domain is composed of seven questions, the knowledge domain is composed of six questions, and the attitude domain is composed of eight questions. The participant’s knowledge has been graded into low, moderate, and high based on the scores they achieved. Less than 50%, 50-75%, and > 75% for low, moderate, and high levels of knowledge, respectively [16].

Study Sampling

The study was a community-based study. The questionnaire has been distributed randomly during the study period using the convenient method of sampling. The chosen distribution list was through popular WhatsApp groups including those from the Ministry of Education and Military and through emails acquired from one of the telecom companies to cover all population classes that fit the inclusion criteria.

Data Analysis

 Data analysis was performed using Statistical Package for the Social Sciences (SPSS ver. 23, IBM Corp., Armonk, NY). Frequency and percentages were used to display categorical variables. Minimum, maximum, mean, and standard deviation were used to present numerical variables. The independent t-test and analysis of variance (ANOVA) test were both used to test for factors associated with knowledge score and attitude score toward organ donation. The ANOVA test was followed by Tukey's post-hoc test to determine where the exact difference between groups exists. The level of significance was set at 0.05.

Ethical considerations

Ethical approval was obtained from the ethical committee in the Jazan region (reference No. REC-43/04/059). All information was kept confidential and was not accessed except for scientific research purposes.

## Results

Sociodemographic characteristics of the study participants

A total of 1019 participants were included in the study. Table 1 shows the sociodemographic profile of the participants. Seven hundred twenty (70.7%) were between 18 and 30 years, 135 (13.2%) were 31-40 years, 129 (12.7%) were between 41 and 50 years, and 35 (3.4%) were older than 50 years. Four hundred three (39.5%) of the participants were males while 616 (60.5%) of them were females. As for education level, seven (0.7%) had a primary school education, 20 (2%) had an intermediate school education, 178 (17.5%) had a high school education, and 814 (79.9%) had a university education. With regards to marital status, 631 (61.9%) of the participants were single while 388 (38.1%) were married. In terms of occupational status, 489 (48%) of the participants were students, 330 (32.4%) were employees, 186 (18.3%) were unemployed, and 14 (1.4%) were retirees. The majority of participants (562; 55.2%) had a monthly income of less than 5000 Saudi Riyal (SR), 202 (19.8%) had an income between 5000 and 10000 SR, 143 (14%) had an income between 10000 and 15000 SR, and 112 (11%) had an income of more than 15000 SR (the average income in this region is 5,000-10,000 SR).

**Table 1:** Social demographic characteristics of study participants

Demographical Characteristics	n	%
Age		
18 - 30 years	720	70.70
31 - 40 years	135	13.20
41 - 50 years	129	12.70
Older than 50 years	35	3.40
Gender		
Male	403	39.50
Female	616	60.50
Educational Level		
Primary school	7	0.70
Intermediate school	20	2.00
High school	178	17.50
University education	814	79.90
Marital Status		
Single	631	61.90
Married	388	38.10
Job		
Student	489	48.00
Employee	330	32.40
Unemployed	186	18.30
Retiree	14	1.40
Residency		
Town	401	39.40
Village	618	60.60
Monthly income		
Less than 5000 SR	562	55.20
5000 - 10000 SR	202	19.80
10000 - 15000 SR	143	14.00
More than 15000 SR	112	11.00

Knowledge responses of study participants

**Figure 1:**
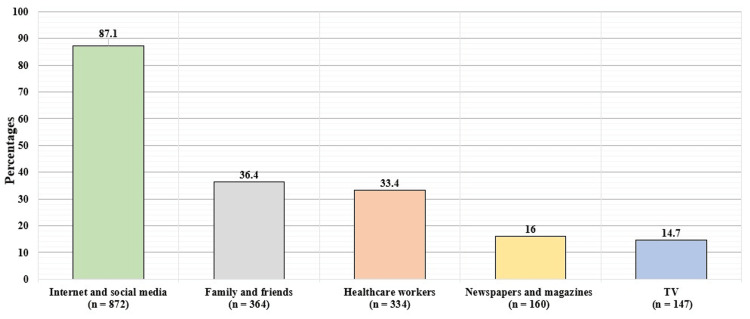
Participants' source of knowledge of organ donation

Table 2 demonstrates the participants' knowledge assessment of organ donation. Almost all of the participants in the survey had heard about organ donation, mainly through the internet or social media (Figure 1). Fifty-nine percent of the participants in the poll believed that organs may be given only after a person's death while 46% of them believed that the person can donate during his life. As for the participant’s thoughts regarding when a person can donate their organs, 471 (46.2%) thought it has to be during the lifetime, 525 (51.5%) thought it was after announcing brain death, 598 (58.7%) thought it was just after death, and 77 (7.6%) reported they didn't know. When they asked which organ can be donated, 925 (90.98%) of participants thought kidneys can be donated, 814 (79.9%) thought only livers can be donated, 707 (69.4%) thought only the heart can be donated, 398 (39.1%) thought eyes can be donated, 521 (51.1%) thought lungs can be donated, and 50 (4.9%) of participants reported they didn’t know what organs can be donated. Seven hundred sixteen (70.3%) reported that they are aware of the presence of centers and official agencies for organ donation in Saudi Arabia. However, more than half of the survey participants were not aware of the regulations governing organ donation.

**Table 2:** Participants' knowledge assessment of organ donation

Questions	n	%
When do you think a person can donate his/her organs? (More than 1 answer can be chosen)		
During lifetime (correct answer)	471	46.2
After announcing brain death (correct answer)	525	51.5
After the death (correct answer)	598	58.7
I don't know	77	7.6
Which of the following organs do you think can be donated? (More than 1 answer can be chosen)		
Kidneys (correct answer)	925	90.8
Liver (correct answer)	814	79.9
Heart (correct answer)	707	69.4
Eyes (correct answer)	398	39.1
Lungs (correct answer)	521	51.1
I don't know	50	4.9
Are you aware of the presence of centers and official agencies for organ donation in Saudi Arabia?		
Yes (correct answer)	716	70.3
No	303	29.7
If you answered the previous question with yes, are you aware of the laws and regulations related to organ donation, brain death, and organ transplantation in Saudi Arabia? (n = 716)		
Yes (correct answer)	327	45.70
No	389	54.30
Knowledge Score (lowest possible score = 0, highest possible score = 11)		
Minimum	0	
Maximum	11	
Mean	6.87	
Standard deviation	2.35	

**Figure 2:**
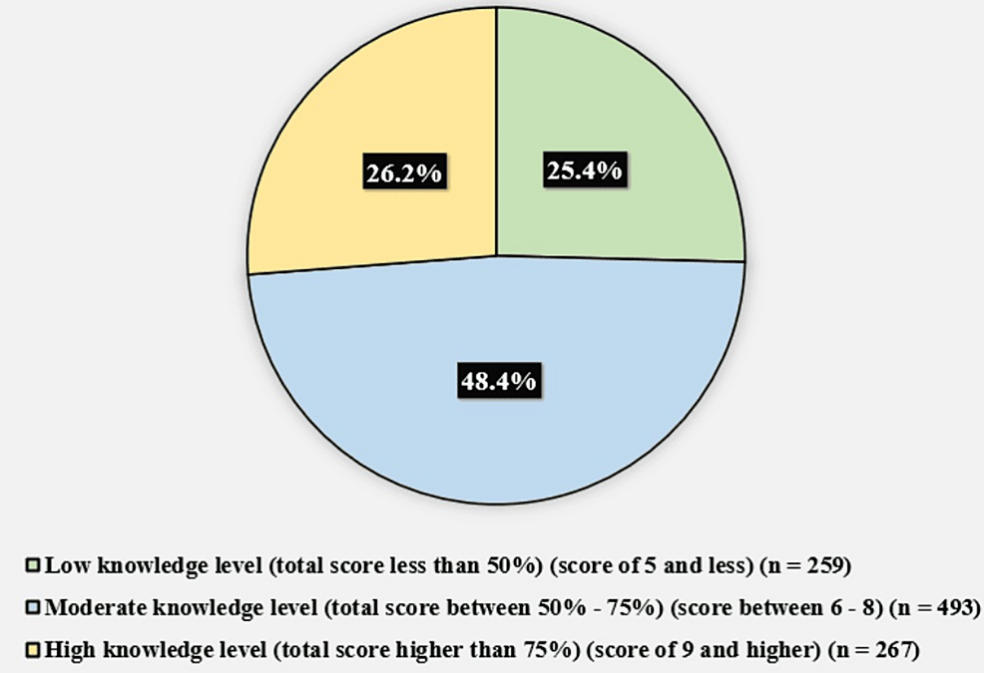
Participants' knowledge levels of organ donation

Figure 2 illustrates the participants' overall knowledge level score toward organ donation. The minimum knowledge score was 0, the maximum was 11, and the mean was 6.87 + 2.35. Two hundred fifty-nine (25.4%) of the participants had a low knowledge level (total score less than 50%) (score of 5 and less), 493 (48.4%) had a moderate knowledge level (total score between 50% and 75%) (score between 6 and 8), and 267 (26.2%) had a high knowledge level (total score higher than 75%) (score of 9 and higher).

Factors associated with knowledge of organ donation

Table 3 presents the factors associated with knowledge of organ donation. Age was significantly associated with knowledge of organ donation (p < 0.001), where it was observed that people aged 18-30 had a higher knowledge score compared to the older age groups. Being an employer was also significantly associated with the knowledge score (p < 0.001). The analysis revealed that students had a significantly higher knowledge score compared to both employees and the unemployed (p < 0.05). Place of residency was also significantly associated with knowledge score (p = 0.03). Those who lived in towns had a higher score than those living in villages (7.14 + 2.18 vs 6.7 + 2.43). Higher education trended toward more knowledge, but this was not statistically significant.

**Table 3:** Factors associated with knowledge of organ donation *Significant at level 0.05

Factor	Knowledge Score toward Organ Donation		P-Value
	Mean	Standard deviation	
Age			< 0.001*
18 - 30 years	7.21	2.21	
31 - 40 years	6.14	2.64	
41 - 50 years	6	2.23	
Older than 50 years	6	2.70	
Gender			0.130
Male	7.01	2.42	
Female	6.78	2.29	
Educational level			0.078
Primary school	5.00	2.58	
Intermediate school	6.25	2.51	
High school	6.75	2.38	
University education	6.93	2.33	
Marital Status			< 0.001*
Single	7.18	2.24	
Married	6.37	2.43	
Job			< 0.001*
Student	7.32	2.18	
Employee	6.42	2.39	
Unemployed	6.59	2.48	
Retiree	5.86	2.51	
Residency			0.003*
Town	7.14	2.18	
Village	6.70	2.43	
Monthly income			0.016*
Less than 5000 SR	7.00	2.31	
5000 - 10000 SR	6.91	2.39	
10000 - 15000 SR	6.30	2.20	
More than 15000 SR	6.89	2.54	

Study participant’s attitude toward organ donation

Table 4 shows the participants' attitude assessment toward organ donation. When the participants were asked about their attitude/perception toward organ donation, 501 (49.2%) of the participants reported being in agreement while 56 (5.5%) were opposed, 121 (11.9%) were unsure, and 341 (33.5%) were neutral. Six hundred ninety-one (67.8%) of the participants reported that if the law and religion encouraged organ donation, they will donate, while 33 (3.2%) reported that they will not. As for those who agreed with organ donation, when they were asked about when they would donate their organs, 36 (7.2%) reported that they would during their lifetime only, 291 (58.1%) reported after their death only, while 174 (34.7%) reported they see no difference in donating during their lifetime or after death.

**Table 4:** Participants' attitude assessment toward organ donation

Question	n	%
What is your attitude/perception of organ donation?		
In agreement with it (positive attitude)	501	49.2
In disagreement with it	56	5.5
I'm not sure	121	11.9
Neutral	341	33.5
Questions Directed to Participants With Agreement Toward Organ Donation (n = 501)		
If you are willing to donate your organs, when would you like to donate your organs? (n = 501)		
During my lifetime only	36	7.2
After my death	291	58.1
I see no difference in donating during my lifetime or after death (positive attitude)	174	34.7
If you are willing to donate your organs, to whom are you willing to donate your organs? (n = 501)		
Only to relatives	46	9.20
Only to non-relatives	4	0.80
To both relatives and non-relatives (positive attitude)	451	90.00
If you are willing to donate your organs, what are your motives? (n = 501)		
Religious motives (positive attitude)	292	58.28
Social motives (positive attitude)	97	19.36
The desire to help others (positive attitude)	405	80.84
Age of the receiver (positive attitude)	81	16.17
The health status of the receiver (positive attitude)	163	32.53
Financial motives	27	5.39
Questions Directed to Participants With Disagreement Toward Organ Donation (n = 56)		
If you are not willing to donate your organs, what are the reasons? (n = 56)		
Because there is no financial benefit	5	8.93
Religious beliefs	22	39.29
Family and social barriers	17	30.36
The fear of not receiving appropriate medical care	13	23.21
The fear of speaking about death	16	28.57
Lack of knowledge about organ donation	11	19.64
Because I think that the organ receiver choice is unfair	12	21.43
Other Attitude Questions (n = 1019)		
If laws and religion encourage donation, will you donate		
Yes (positive attitude)	691	67.8
No	33	3.2
I'm not sure	295	28.9
What is your personal attitude /perception toward promoting organ donation?		
I think we need it (a positive attitude)	562	55.20
I think we don't need it	100	9.80
I'm not sure	357	35.00
If you think there is no need for the promotion of organ donation, what are the reasons?		
Because it may lead to organ trafficking	47	47.00
The fear of wasting the donated organs by losing them or inappropriate handling of it	15	15.00
Religious beliefs	10	10.00
Other reasons (like the post-operative pain, the scars and body disfigurement, and fear of family abandonment)	22	22.00
No reasons were specified	6	6.00
Positive Attitude Score (lowest possible score = 0, highest possible score = 10)		
Minimum	0	
Maximum	10	
Mean	3.35	
Standard deviation	2.90	

Among those who were willing to donate, when they asked to whom they will donate their organs, 46 (9.2%) reported that they would donate only to their relatives, four (0.8%) reported only to non-relatives, while 451 (90.0%) reported to both relatives and non-relatives. As to what motivated the participants to donate their organs, the most commonly reported motives were the desire to help others in 405 (80.8%), followed by religious motives in 292 (58.3%), and finally the health status of the receiver in 163 (32.5%). The reasons for opposing organ donations included religious beliefs (39.29%), family and social barriers (30.36%), and fear of speaking about death (28.57%).

Most participants felt that organ donations should be promoted (55.2%). The most commonly reported cause for thinking that promoting donation is not needed/important was thinking it may lead to organ trafficking for 47 (47.0%). The minimum positive attitude score was 0, the maximum was 10, and the mean was 3.35 + 2.9.

**Figure 3:**
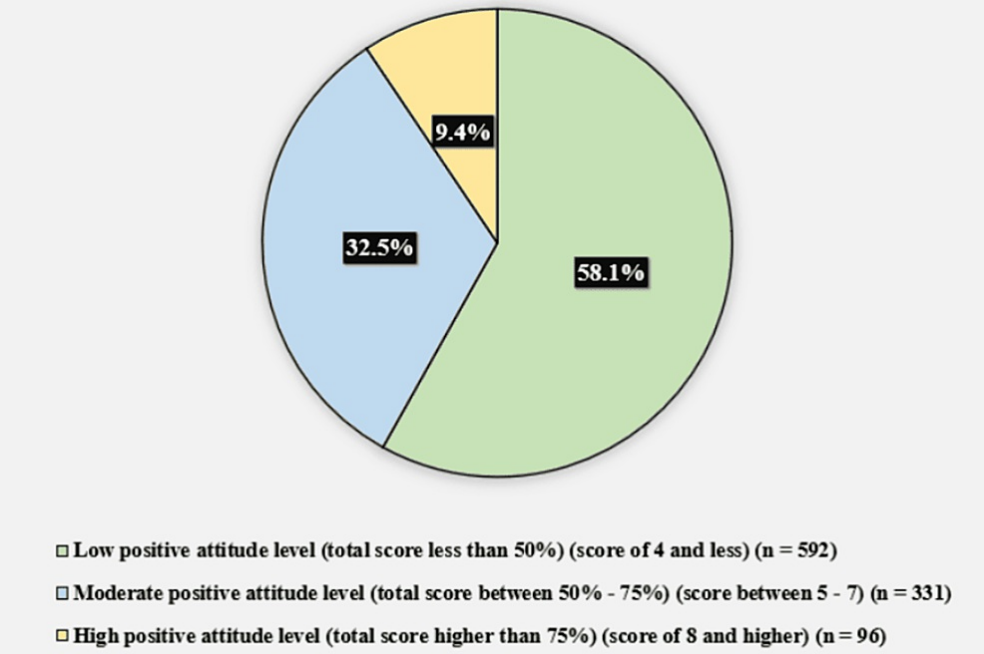
Participants' attitude levels toward organ donation

Figure 3 displays the participants' attitude level toward organ donation. Five hundred ninety-two (58.1%) of the participants had a low positive attitude level (total score less than 50%) (score of 4 and less), 331 (32.5%) had a moderately positive attitude level (total score between 50% - 75%) (score between 5 and 7), and 96 (9.4%) had a high positive attitude level (total score higher than 75%) (score of 8 and higher).

Factors associated with a positive attitude toward organ donation

Table 5 demonstrates the factors associated with a positive attitude toward organ donation. Younger age was significantly associated with a positive attitude toward organ donation (p = 0.028). The analysis revealed that single participants had a significantly higher positive attitude score compared to married participants (3.55 + 2.98 vs 3.03 + 2.75). Being employed was also significantly associated with a positive attitude score (p = 0.003). Tukey's post-hoc test revealed that students had a significantly higher positive attitude score compared to unemployed (p < 0.05). Gender, education level, place of residency, and monthly income were all not significantly associated with a positive attitude score.

**Table 5:** Factors associated with a positive attitude toward organ donation *Significant at level 0.05

Factor	Positive Attitude Score Toward Organ Donation		P-value
	Mean	Standard deviation	
Age			0.028*
18 - 30 years	3.52	2.99	
31 - 40 years	2.79	2.73	
41 - 50 years	3.02	2.49	
Older than 50 years	3.4	2.97	
Gender			0.802
Male	3.33	2.94	
Female	3.37	2.88	
Educational level			0.634
Primary school	3.71	1.60	
Intermediate school	3.30	2.49	
High school	3.60	3.03	
University education	3.30	2.89	
Marital status			0.005*
Single	3.55	2.98	
Married	3.03	2.75	
Job			0.003*
Student	3.65	3.01	
Employee	3.28	2.76	
Unemployed	2.75	2.78	
Retiree	2.79	2.91	
Residency			0.735
Town	3.39	2.87	
Village	3.33	2.93	
Monthly income			0.750
Less than 5000 SR	3.38	2.97	
5000 - 10000 SR	3.41	2.88	
10000 - 15000 SR	3.11	2.82	
More than 15000 SR	3.45	2.72	

## Discussion

The study assessed the knowledge and attitudes toward organ donation in Jazan, Saudi Arabia. The majority of study participants were young (18-30 years) and students. In addition, 80% of them have a university education or higher.

Specifically, our data demonstrated that the media was the most effective source of information about organ donation, whereas schools were the least effective source. Our findings are consistent with those of research conducted in India, in which around 61% of participants learned about the study via mainstream media [17]. As a result, a media campaign may have a major impact on organ donation awareness and beliefs, as evidenced by research in which post-media surveys revealed considerable improvements in organ donation awareness.

Our study also showed that participants are aware of when someone can donate their organs. Their answers were relatively similar, in that 59%, 51%, and 46% of them answered after death, after brain death, and during people’s lives, respectively. This finding is consistent with a study conducted among the eastern Moroccan population where more than 90% of study participants knew that organ donations can come from both cadaveric and living persons [18]. However, Taimur et al. showed that only a minority of the study participants were aware of the possibility of donation during a person’s lifetime [16]. Likewise, a local study in Al-Kharj showed similar findings in terms of the participant’s awareness about when to donate the organs [13]. This difference in the level of awareness of organ donation timing is varied according to culture, educational level, the provided health care services, and campaign efforts [13,19].

The kidney was the most common organ that can be donated according to many surveys. This is consistent with our study, which showed that 91% of study participants believed that the kidneys are the most common organ that can be donated followed by the liver and heart. The population's choice of kidneys is somewhat expected as there is an increased awareness in Saudi Arabia of kidney donation because of the expanded number of kidney failure patients and particularly in Jazan [6,13,17,19].

Providing the fact that the Saudi Center for Organ Transplantation (SCOT) has been a successful center for practically all forms of organ transplantation since its inception, the present research revealed that 70% of the study population was aware of the presence of an organ donor registration facility in Saudi Arabia [12]. This finding is higher than the awareness level of the Moroccan study population, which showed that 51% were aware of the existence of these centers [19]. Disappointingly, more than 50% who are aware of the presence of a donation center in the present study were not aware of its regulations; efforts need to be augmented to inform the public about the policy and procedures of these centers.

In general, our study population showed a moderate level of knowledge of organ donation, in which 48% scored 6-8 out of 11 points (mean of 6.86 and SD 2.35). This finding can be explained by the fact that most of the participants are young and have attained a university level of education or higher, in which access to information is readily available.

Our study also found that young people, those who have a job, and people living in cities are more likely to be knowledgeable as compared to their counterparts. People who are living in cities are closer to the health facilities and more aware of the educational programs conducted in these facilities. Therefore, it is expected that they know more about the provided health care services compared to village residents. Interestingly, our study found no difference in the knowledge level among participants with different educational levels. This finding comes in concordance with a similar national survey performed in Jeddah and in contrast to a Korean study conducted by Kim et al., which showed a higher level of organ donation level is associated with a higher educational level, particularly among health care professionals [20-21].

In the present study, around 50% of study participants had a positive attitude towards organ donation. This percentage has increased to 68% if the laws and religion encourage the donation. Fifty-eight percent of participants are willing to donate after death. Additionally, 34% of them see that there is no difference in the timing of donation, whether during life or after death. On the other hand, only 5.5% of study participants exhibited a negative attitude towards organ donation. This is in contrast to a similar study conducted in Saudi Arabia and Nigeria, which showed that around 28% and 40% of participants, respectively, were refused organ donation, which is about five times the percentage in the present study [20,22]. The reason cannot be extrapolated from the study findings but it seems that the desire of the population to decrease the burden of organ failures and help others in the region is the likely explanation. It is also notable that 90% of participants were willing to donate an organ to anyone and not just to relatives. This could reflect the altruistic behavior of the study participants. This is in contrast to other studies in other regions of Saudi Arabia conducted by Agrawal and Al-Harthi et al., which showed that 75% and 60% of the general population, respectively, and 48.8% in Morocco have refused organ donation [13,19,23].

Among patients with a positive attitude toward organ donation, this study showed that the absence of incentives doesn’t largely influence the decision to donate (5.4%). Furthermore, the monthly income wasn’t statistically associated with a significant difference in participants’ attitudes. This finding is contrary to a similar local study that showed that a lack of incentives could represent a barrier to being a donor [19]. The presence of such a background among the present study participants (94%) would serve as a potential scaffold to inform the public about the importance of organ donation and expedite its process. It could also help organizations and campaigns to target such a population by increasing the transplantations of organs in this community.

Even though the vast majority of Saudi Arabia's population is Muslim, this survey found that just 39% of the people viewed religion as a barrier to organ donation and refused to donate on this basis. Similarly, research done at the Dhahran Military Hospital revealed that 68.6% of the participants believed it was lawful to donate organs as compared to 26.2% who believed it was banned in Islamic tradition [24]. Another research conducted in Kuala Lumpur revealed that just 10% of those who answered the survey declined organ donation due to their religious beliefs [25-26]. As a result, as individuals gain more education, the religious barrier becomes less and less of an actual barrier. On the contrary, it is seen as saving thousands of lives and assisting others, which is in accordance with Islamic principles of aiding others [27]. The Islamic religion encourages people to save lives and support each other as stated clearly in the Holy Quran and Sunna [27-29]. Organ donation-related obstacles were investigated in research that focused mostly on Catholic Christians. The findings found that 17% of respondents declined to contribute because of their religious views while 10% claimed that religion did not play a part in their decision-making [30].

The biggest proportion of participants (47%) agreed that their corpses should be preserved after death, mainly because of the fear of trafficking caused by organ donation. This is similar to the findings of research that found that 16.7% of participants want their remains to be preserved after death [20,22]. Many participants in this survey were opposed to organ donation for a variety of reasons, including the fact that 22% are afraid of well-known complications like postoperative pain, scars and body disfigurement, and fear of family abandonment [19,20,23]. Therefore, setting regulations that will maintain the donor’s health during their lives and provide easy access to follow-up to health care facilities, whenever needed, will help in encouraging the general population to donate their organs. Of note, the government of Saudi Arabia, as part of its role in encouraging and facilitating the donation process to the community, has given many incentives to the donors. This includes but is not limited to offering the third degree of King Abdelaziz Medal to all donors, 50% discounts on national flights, and 50000 Saudi riyals given in full right after the donation. Moreover, the government provides access cards to donors as part of a donor incentive program to facilitate their banking and traveling services. People who donate their organs will also have full-time access to health care facilities for checkups and health maintenance [12].

Aside from the religious beliefs and the fear of complications, there were three other factors identified in this study that make the participants not willing for or promoting donation: family and social barriers, the fear of speaking about death, lack of knowledge about donation, and the unfairness of organ receiver choice. These barriers have also been identified in different studies at local and international levels [14,17,19].

The study has several strengths that are worth acknowledging. Although it is not the first one to be conducted in Saudi Arabia, it is the first one addressing this issue in the Jazan region where health care, educational services, and campaigns are evolving to catch up with other regions in the kingdom [5]. The sample size of surveyed participants is considered the largest compared to other national studies [14,17,28]. Additionally, the online questionnaire used in this study has the advantage of avoiding the interviewer’s bias, which may affect the validity of the results. The population of interest in the present study was the general population, which is the class of people who ultimately need to be aware and educated about the organ donation process in contrast to medical school students who were the target population in most similar studies despite their higher rate of education exposure.

Finally, the scorning method that has been used in the current research to categorize the participant’s response levels in both knowledge and attitude domains into low, intermediate, and high has helped in understanding the stratification of study participants. Nevertheless, many limitations need to be addressed. First, the convenient method of sampling may make the results not generalizable. Despite its non-generalizability, the current study showed the current positive situation towards organ donation among this specific region population. Second, some other variables were not included in the current questionnaire such as the ability of the donors to change their decisions after they give their permission to donate, the participant’s knowledge about the current status of patients with organ failures, and the waiting list in the country. However, the items in the questionnaire were validated and piloted before its implementation. Moreover, the questionnaire items have addressed many important themes that cover the study objectives.

## Conclusions

This study showed a moderate amount of knowledge and a 50% positive attitude among participants toward organ donation. It also showed that the level of education and lack of incentives were not significant factors to affect the individual's knowledge and perception when they decided to donate organs. This opportunity should be used appropriately and encourage the campaigns and high authorities to focus on increasing the donation among this specific population and shorten the waiting time in the transplantation pool. Moreover, further studies across the kingdom are needed to highlight the reasons for variations in knowledge and attitude among the Saudi population. Information regarding organ donation should be incorporated with clear messages in various mass media.
